# A Novel Computed Tomographic Angiography Tortuosity Index to Predict Successful Sentinel Cerebral Embolic Protection Delivery for Transcatheter Aortic Valve Replacement

**DOI:** 10.1016/j.shj.2022.100021

**Published:** 2022-03-31

**Authors:** Denny Wang, Craig Basman, Sahar Mahani, Arber Kodra, Luigi Pirelli, Priti Mehla, Nirav Patel, Jacob Scheinerman, Nirmay Bhanderi, Chad Kliger

**Affiliations:** Department of Cardiovascular & Thoracic Surgery, Lenox Hill Hospital/Northwell Health, New York, New York, USA

**Keywords:** Computed tomographic angiography, Embolic protection, Modeling, Tortuosity, Transcatheter aortic valve replacement

## Abstract

**Background:**

Percutaneous transradial placement of the Sentinel cerebral embolic protection device (CEPD) (Boston Scientific) is indicated during transcatheter aortic valve replacement to capture embolic material in patients without excessive tortuosity of the right subclavian/innominate arteries. We aimed to generate a quantitative tortuosity index (TI) from the preoperative computed tomographic angiography (CTA) as an objective measure of tortuosity to determine suitability for CEPD placement.

**Methods:**

Eighty-one patients considered for CEPD were included in this study. A centerline of the right subclavian/innominate arteries was generated from preoperative CTA scans. Three-dimensional Cartesian coordinates of landmarks along the centerline were used to calculate curvature. Tortuosity was derived as a change in angulation along each vessel segment. Peak and average TI values were calculated.

**Results:**

Sixty-seven patients had CEPD placement attempted. Unsuccessful CEPD placement occurred in 3 of 67 (4.4%) patients. The peak tortuosity for the successful, unsuccessful, and visually tortuous (not attempted) cohorts were 49.66 ± 11.96°/cm, 113.92 ± 5.70°/cm, and 70.44 ± 17.01°/cm, respectively. The peak and average TI of the successful cohort follows a normal distribution. A proposed TI cutoff for safe CEPD was peak tortuosity of 74°/cm and average tortuosity of 30°/cm, 2 standard deviations above the peak and average TI of the successful cohort. All unsuccessful CEPD patients fell outside the boundaries. Half of the visually tortuous patients were within the boundaries but did not have CEPD attempted.

**Conclusions:**

A novel TI based on preoperative CTA can assist in selecting patients for transradial CEPD. Our proposed quantitative tool may help to appropriately include and exclude patients for CEPD placement.

## Introduction

Despite significant advances in the field of structural heart disease, stroke continues to be a major complication associated with transcatheter aortic valve replacement (TAVR).^1^ To potentially reduce this risk, the use of a cerebral embolic protection device (CEPD) can be deployed to capture or deflect embolized material. The Sentinel CEPD (Boston Scientific) is the first and only Food and Drug Administration–approved filter device to use during TAVR to reduce the risk of stroke.^2^ The device is implanted via a right radial artery approach and has 2 filters that are deployed within the brachiocephalic and left common carotid arteries. Extreme vessel tortuosity is an exclusion criterion for device implantation. Currently tortuosity is analyzed qualitatively. This method of assessment can introduce significant interobserver variability. Although there are several metrics used to assess vessel tortuosity, there exists no standardized method to determine anatomic suitability for CEPD.^3^, ^4^, ^5^, ^6^, ^7^, ^8^, ^9^, ^10^ One of the most common methods measures the ratio between the total vessel length and the end-to-end distance. A weakness of this method is its inability to detect regions of high tortuosity. Therefore, we aimed to generate a quantitative tortuosity index (TI) from the preoperative TAVR computed tomography angiography (CTA) as an objective measure of both peak and average tortuosity to determine suitability for CEPD placement.

## Methods

From November 2018 to March 2020, 81 consecutive patients at Lenox Hill Heart and Lung, Northwell Health, who underwent structural heart procedures with planned CEPD placement were included for analysis. Patients found to have significant stenosis or heavy calcification of the left subclavian or innominate arteries were excluded. All patients had preoperative cardiac CTA (256-slice iCT scanner, Philips Healthcare, Cleveland, Ohio) to include the great vessel and proximal upper extremity vasculature using helical scan mode with multiphase acquisition (16 phases, 6.25% RR interval increments) and electrocardiogram gating. The minimum vessel diameter and the calcification of the right subclavian and innominate arteries were also assessed on the two-dimensional (2D) multiplanar reconstruction views. This prospective study was approved by the Northwell Health Institutional Review Board. No informed consent was required.

**Figure 1:**
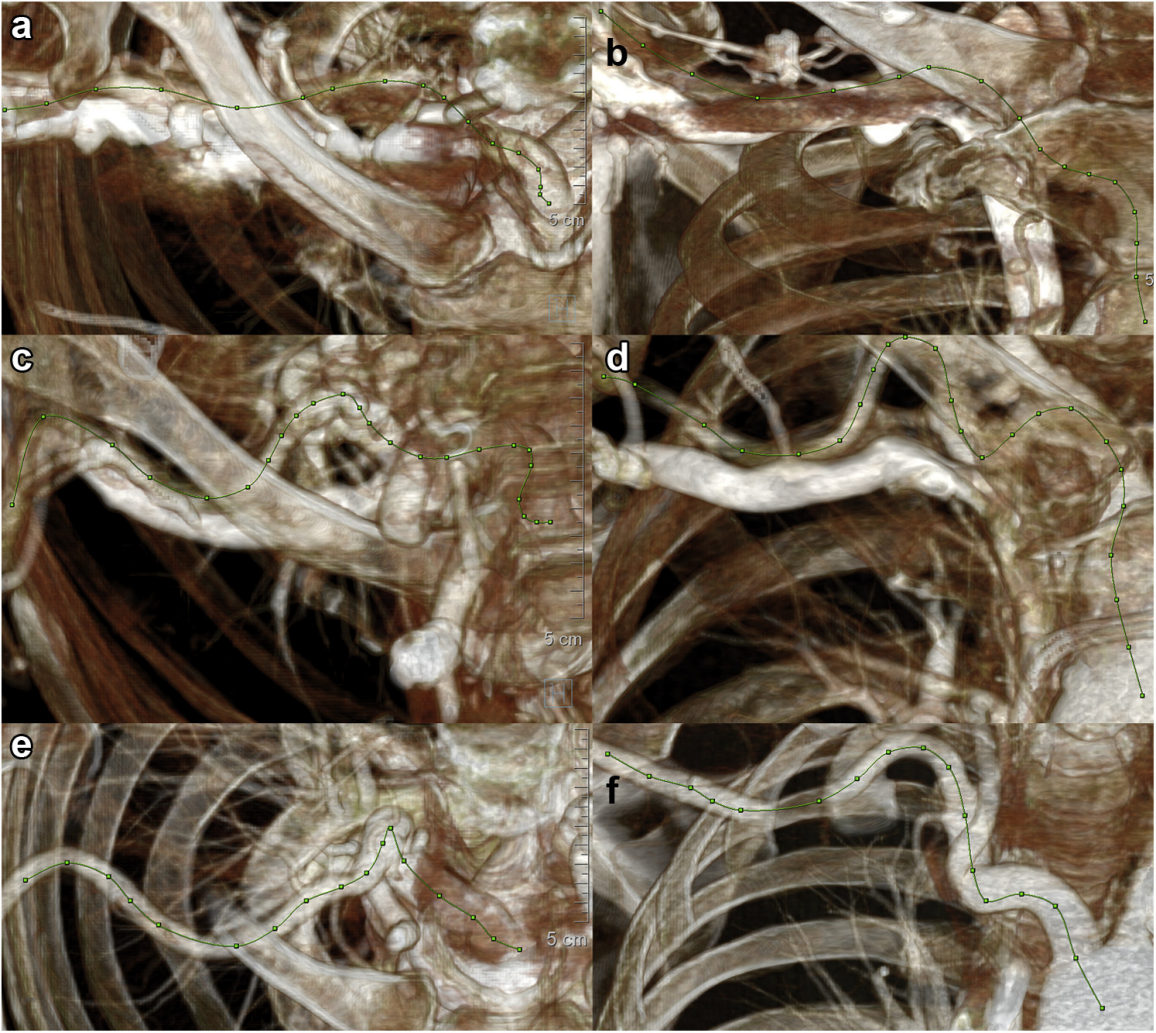
**Using the automated Thoracic Endovascular Aortic Repair tool and/or manual polyline tool (SyngoX software, Siemens Healthineers, Erlangen, Germany), a centerline was generated from the distal right axillary, through the subclavian/brachiocephalic arteries, down to the aortic arch.** Nodes were placed approximately 1 cm along the length. Two snapshots were then performed in the coronal (a) and axial (b) planes to obtain the x, y, and z coordinates of each node as shown. (a) and (b) show the centerline of a patient from the successful cohort with peak/average tortuosity of 27.48°/cm and 14.56°/cm, respectively. (c) and (d) are coronal and axial views of the centerline of a patient from the unsuccessful cohort with a peak/average tortuosity of 108.79°/cm and 34.89°/cm, both above the maximum cutoff values of 74°/cm and 30°/cm, respectively. (e) and (f) are coronal and axial views of the centerline of a patient from the visually tortuous cohort with a peak/average tortuosity of 47.66°/cm and 22.70°/cm, respectively.

On the 75% phase image and using the automated Thoracic Endovascular Aortic Repair tool and/or manual polyline tool (SyngoX software, Siemens Healthineers, Erlangen, Germany), a centerline was generated from the distal right axillary, through the subclavian/brachiocephalic arteries, down into the aortic arch. Nodes were placed approximately 1 ​cm along the centerline to serve as markers. Adjustments to the nodes could be made as needed in the 2D multiplanar reconstruction view to ensure the nodes are located within the center of the vessel. Two snapshot images, one anteroposterior/coronal (x, y coordinates) and a second axial at the same magnification (x, z coordinates), were taken on the three-dimensional (3D) volume rendered view of the vessel with centerline to scale (Figure 1). The 3D Cartesian coordinates of the nodes, and the relative distance between them were determined based on the pixel location of the nodes on the 3D volume rendered view (Supplemental Table 1c provides an example of pixel coordinates obtained from the axial snapshot, Supplemental Figure 1a, and the coronal snapshot, Supplemental Figure 1b).

**Figure 2:**
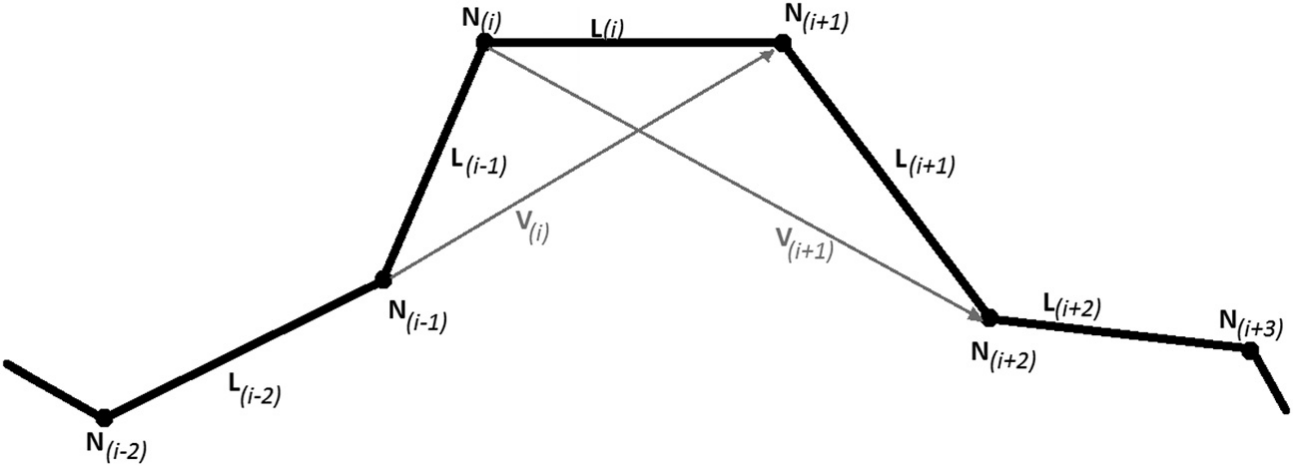
**Centerline model is made of node and line segments.** Tortuosity of the vessel is computed from this model. Tortuosity at segment L(i) is found by dividing the angle between vector V(i) and V(i+1) by the distance between node N(i) and N(i+1).

To approximate the curve, straight line segments were used to connect successive nodes; nodes and line segments make up the centerline model. The tortuosity of the vessel was computed from this model (Figure 2). Measurement of tortuosity was derived from the definition of curvature. Curvature, k, for a specific point along a continuous curve is defined as

(1) $$k=\;\frac{\partial \vec{T}}{\partial s}$$

where ∂T is the differential change in the unit tangent vector at a specified point and ∂s is the differential change in arc length. This equation is not directly applicable to the centerline model as the position of the curve for the centerline model is not explicitly defined by parametric functions. A modified discrete form of the curvature equation is required. Instead of tortuosity being determined at a point, tortuosity will be determined over a discrete line segment.

Tortuosity measure of a specific line segment is defined as the change in angle divided by the length of that segment (Figure 2). Our model consists of n nodes separated by n−1 line segments. For the generic line segment L(i), the length can be directly calculated as the coordinates of the nodes at either end are known. The scale included in the snapshot is used to convert the calculated segment length from pixels to centimeters. For the line segment L(i), the change in angle can be estimated as the angle between vector V(i) and vector V(i+1). Vector V(i) points from node N(i−1) to node N(i+1). Vector V(i+1) points from node N(i) to node N(i+2). Since the coordinates of all these nodes are known, the angle between the 2 vectors can also be calculated. These calculations can be summarized in the following equation

(2) $$k\left(i\right)=\frac{\cos^{-1}\left(\frac{\vec{V_{i}}\;\cdot \vec{V_{i+1}}}{\left\|\vec{V_{i}}\right\|\left\|\vec{V_{i+1}}\right\|}\right)}{\sqrt{{\left(X_{i+1}-X_{i}\right)}^{2}+{\left(X_{i+1}-Y_{i}\right)}^{2}+{\left(Z_{i+1}-Z_{i}\right)}^{2}}}$$

where k(i) is the tortuosity at line segment L(i). X(i), Y(i), and Z(i) are the x, y, and z coordinates of node N(i) respectively.

To automate this task, a script was written in Visual Basic for Applications in Microsoft Excel to perform the calculations. The known 3D coordinates of the nodes with the scale were entered into Microsoft Excel 2013 (Microsoft, Redmond, Washington), and the tortuosity was calculated for all line segments except for the first and the last segments. The average tortuosity of the centerline model was also calculated by weighting the tortuosity of each line segment based on its length and averaging the values. The first and last segments are excluded from the average tortuosity as they do not have a tortuosity value.

(3) $$\mathrm{average}\;\mathrm{tortuosity}=\frac{\sum_{i=2}^{n-2}k\left(i\right)\sqrt{{\left(X_{i+1}-X_{i}\right)}^{2}+{\left(X_{i+1}-Y_{i}\right)}^{2}+{\left(Z_{i+1}-Z_{i}\right)}^{2}}}{\sum_{i=2}^{n-2}\sqrt{{\left(X_{i+1}-X_{i}\right)}^{2}+{\left(X_{i+1}-Y_{i}\right)}^{2}+{\left(Z_{i+1}-Z_{i}\right)}^{2}}}\;$$

Intraobserver variability was assessed by asking one reader to evaluate the same snapshots of the subclavian/brachiocephalic artery twice. Interobserver variability was assessed by comparing the reads from different readers. Variability was assessed using Bland-Altman plots. To assess reliability, interclass correlation coefficients (ICCs) were calculated for all combinations of readers and TI.

For patient characteristics and results, continuous variables are reported as mean with standard deviation (SD) and categorical variables are presented as percentages. *P*-values <0.05 were considered statistically significant. All statistical analyses were performed with Microsoft Excel and Prism 9.2.0 (GraphPad Software, San Diego, California).

## Results

**Figure 3:**
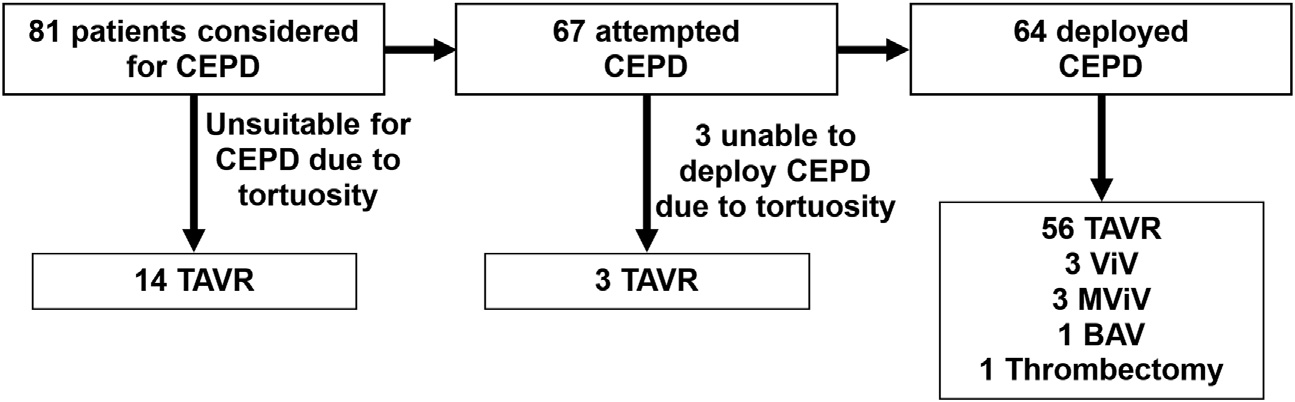
**Study workflow showing the screening of patients considered for cerebral embolic protection device (CEPD) placement.** Three cohorts of patients included patients that were deemed unsuitable due to visual tortuosity (Visually Tortuous), unable to deploy (Unsuccessful), and able to deploy (Successful). Types of interventions are noted: transcatheter aortic valve replacement (TAVR), aortic Valve-in-Valve (ViV), mitral Valve-in-Valve (MViV), balloon aortic valvuloplasty (BAV), and pulmonary thrombectomy.

Eighty-one patients (mean age: 78 ± 8.8 years, 56.8% female, 20.0% bovine variant) were analyzed. Fourteen patients had vessels deemed visually tortuous, and CEPD was not therefore attempted. CEPD was attempted in 67 patients. Successful placement of the CEPD occurred in 64 of 67 patients (95.6%) (Figure 3). Baseline patient characteristics are summarized in Table 1. The majority of these patients underwent TAVR (90.1%). The average minimum vessel diameter of the right subclavian and innominate artery was approximately 5.5 mm for all groups. The smallest minimum vessel diameter measured is 3.59 mm, large enough to accommodate the 6F profile of the sentinel CEPD. Using one-way analysis of variance, there was no significant difference in the minimum vessel diameters for all groups (*p* = 0.97). Combining all 3 groups, 73 of 81 (90.1%) patients had no or mild calcification of the right subclavian and/or innominate arteries, while 8 of 81 (8.9%) patients had a moderate degree. There were no strokes or vascular complications related to deployment of CEPD in any cohort. For the successful cohort, the values of peak and average TI were 49.66°/cm ± 11.96°/cm and 21.19°/cm ± 4.33°/cm, respectively.

**Table 1:** Baseline characteristics and endpoints of the 3 patient cohorts

Baseline characteristics	Successful	Unsuccessful	Visually tortuous
	(n = 64)	(n = 3)	(n = 14)
Age ± SD (y)	76.8 ± 8.7	82.4 ± 6.6	83.3 ± 7.8
Gender (Female)	38 (59.3%)	1 (33.3%)	7 (50.0%)
Bovine arch type	15 (23.4%)	0 (0%)	1(7.1%)
Indication for procedure:			
TAVR	56 (86.1%)	3(100%)	14(100%)
Aortic Valve-in-Valve	3 (4.7%)	0 (0%)	0 (0%)
Mitral Valve-in-Valve	3 (4.7%)	0 (0%)	0 (0%)
Balloon valvuloplasty	1 (1.6%)	0 (0%)	0 (0%)
Pulmonary thrombectomy	1 (1.6%)	0 (0%)	0 (0%)
Prewiring of the brachiocephalic/left common carotid	10 (15.6%)	0 (0.0%)	-
Minimum vessel diameter of the right subclavian/innominate artery ± SD (mm)	5.6 ± 1.0	5.5 ± 1.2	5.5 ± 0.6
Right subclavian/innominate calcification:			
None	31 (48.4%)	2 (66.6%)	7 (50.0%)
Mild	27 (42.2%)	0 (0%)	6 (42.9%)
Moderate	6 (9.4%)	1 (33.3%)	1 (7.1%)
Severe	0 (0%)	0 (0%)	0 (0%)
TIA/strokes	0 (0%)	0 (0%)	0 (0%)
Vascular complications related to CEPD	0 (0%)	0 (0%)	0 (0%)

Abbreviations: CEPD, cerebral embolic protection device; SD, standard deviation; TAVR, transcatheter aortic valve replacement; TIA, transient ischemic attack.

For the unsuccessful cohort, the peak and average tortuosity were 113.92 ± 5.71°/cm and 27.93 ± 6.42°/cm, respectively. For the visually tortuous cohort, the peak and average tortuosity were 70.45 ± 17.01°/cm and 27.38 ± 4.31°/cm, respectively.

Using one-way analysis of variance, there was a significant difference within the 3 cohorts for both peak TI (*p* < 0.01) and average TI (*p* < 0.01). Post hoc analysis using Tukey’s test showed a significant difference in the peak TI for all 3 cohorts (all adjusted *p* < 0.01). A significant difference was also noted for the average TI for the successful cohort compared to both the unsuccessful cohort (adjusted *p* = 0.03) and the visually tortuous cohort (adjusted *p* < 0.01). There was no difference detected between the unsuccessful cohort and the visually tortuous cohort for the average tortuosity index (adjusted *p* = 0.98).

**Figure 4:**
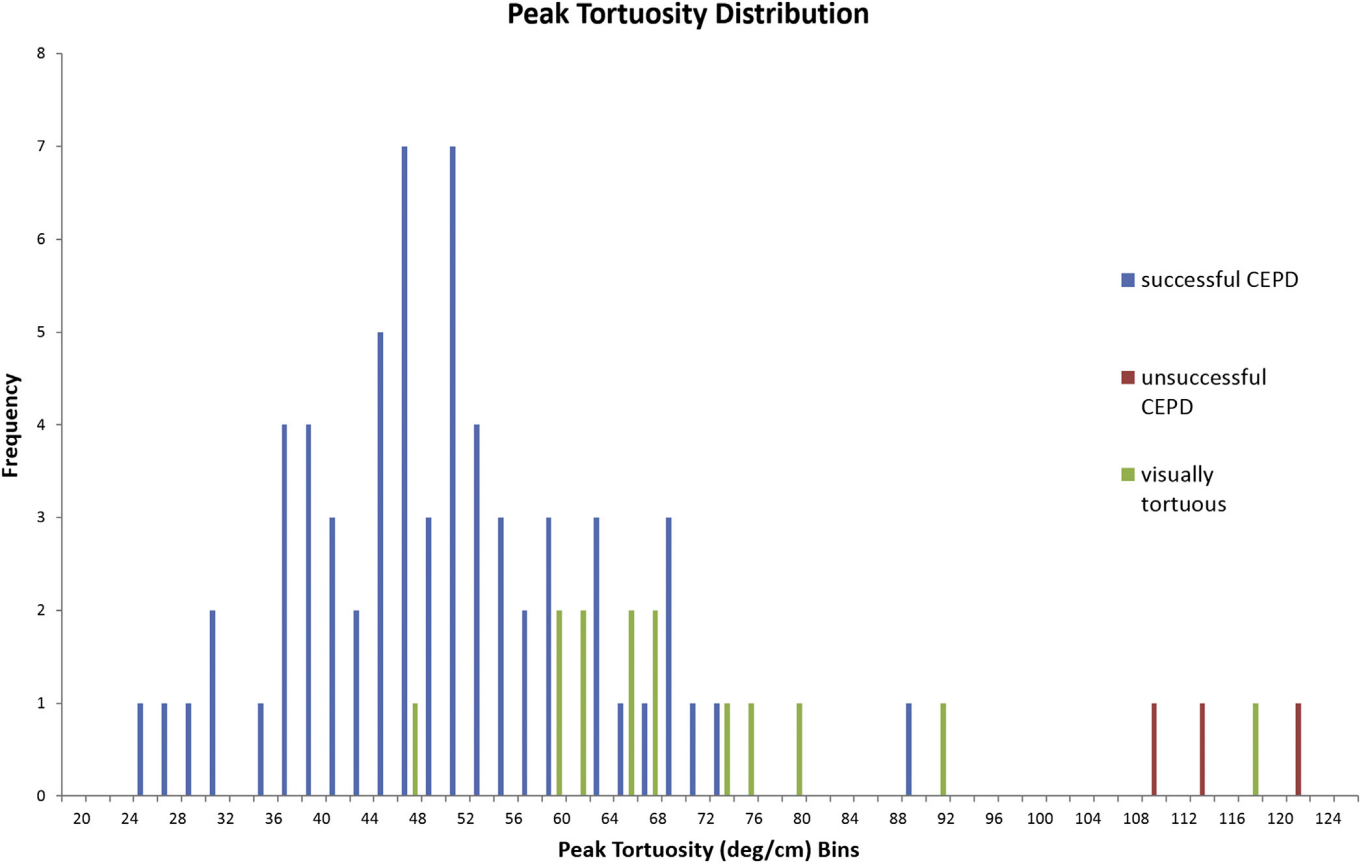
**Graph of peak tortuosity for each patient is shown.** Excluding one outlier, all members of the successful cohort were below the cutoff of a peak tortuosity of 74°/cm, 2 standard deviations above the mean of the successful cohort. Abbreviation: CEPD, cerebral embolic protection device.

**Figure 5:**
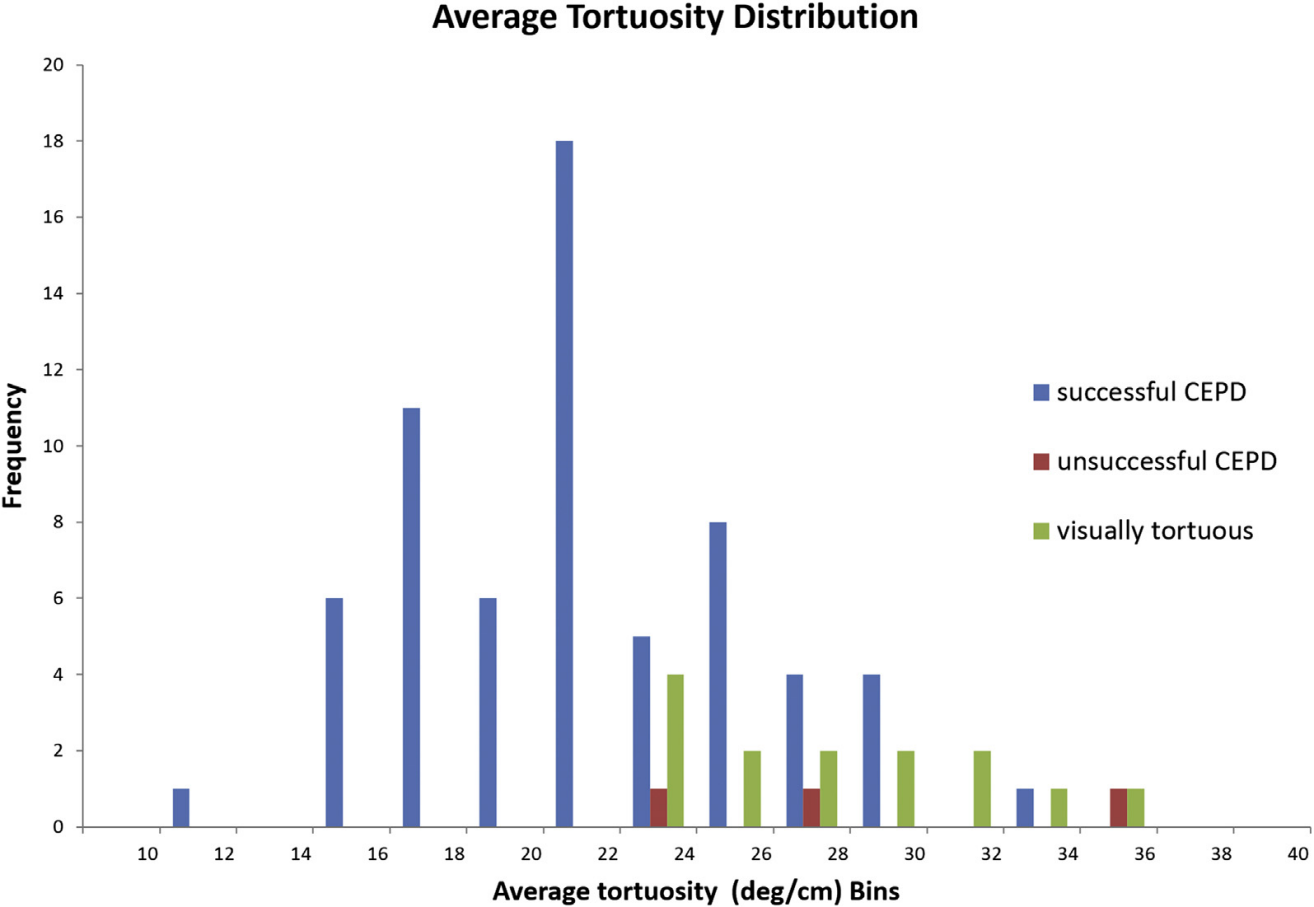
**Graph of average tortuosity for each patient is shown.** Excluding one outlier, all members of the successful cohort were below the cutoff of an average tortuosity of 30°/cm, 2 standard deviations above the mean of the successful cohort. Abbreviation: CEPD, cerebral embolic protection device.

**Figure 6:**
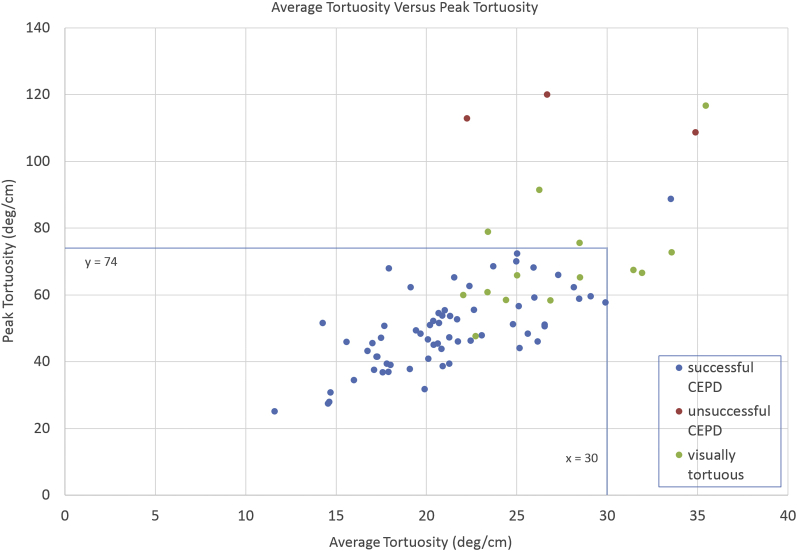
**Scatterplot of peak vs. average tortuosity is shown.** Cutoff values for peak and average tortuosity are noted (blue lines) at 74°/cm and 30°/cm, respectively. All patients with unsuccessful CEPD delivery fall outside the boundaries identified. However, nearly one-half of patients who were deemed visually too tortuous by preoperative CTA were within the margins but did not have CEPD placement attempted. Abbreviations: CEPD, cerebral embolic protection device; CTA, computed tomographic angiography.

Histograms showing the distribution of values for the peak and average TI and a scatterplot of peak vs. average TI are shown in Figure 4, Figure 5, Figure 6. The range of values of both the peak TI and average TI for the successful cohort followed a normal distribution as determined with the Shapiro-Wilk test (*p* = 0.37, *p* = 0.43). Ninety-five percent of all data points for the successful cohort were within approximately 2 SDs of the mean value of the peak and average TI. The upper limit was approximately 74°/cm for the peak TI and 30°/cm for the average TI. These upper limits were used as a cutoff for safe deployment of CEPD and are displayed on Figure 6. All patients of the unsuccessful cohort fell outside the boundaries of the peak/average tortuosity cutoffs. However, one-half of the visually tortuous patients (7/14, 50.0%) were within the safe cutoff margins, but did not have CEPD placement attempted (Figure 6).

**Figure 7:**
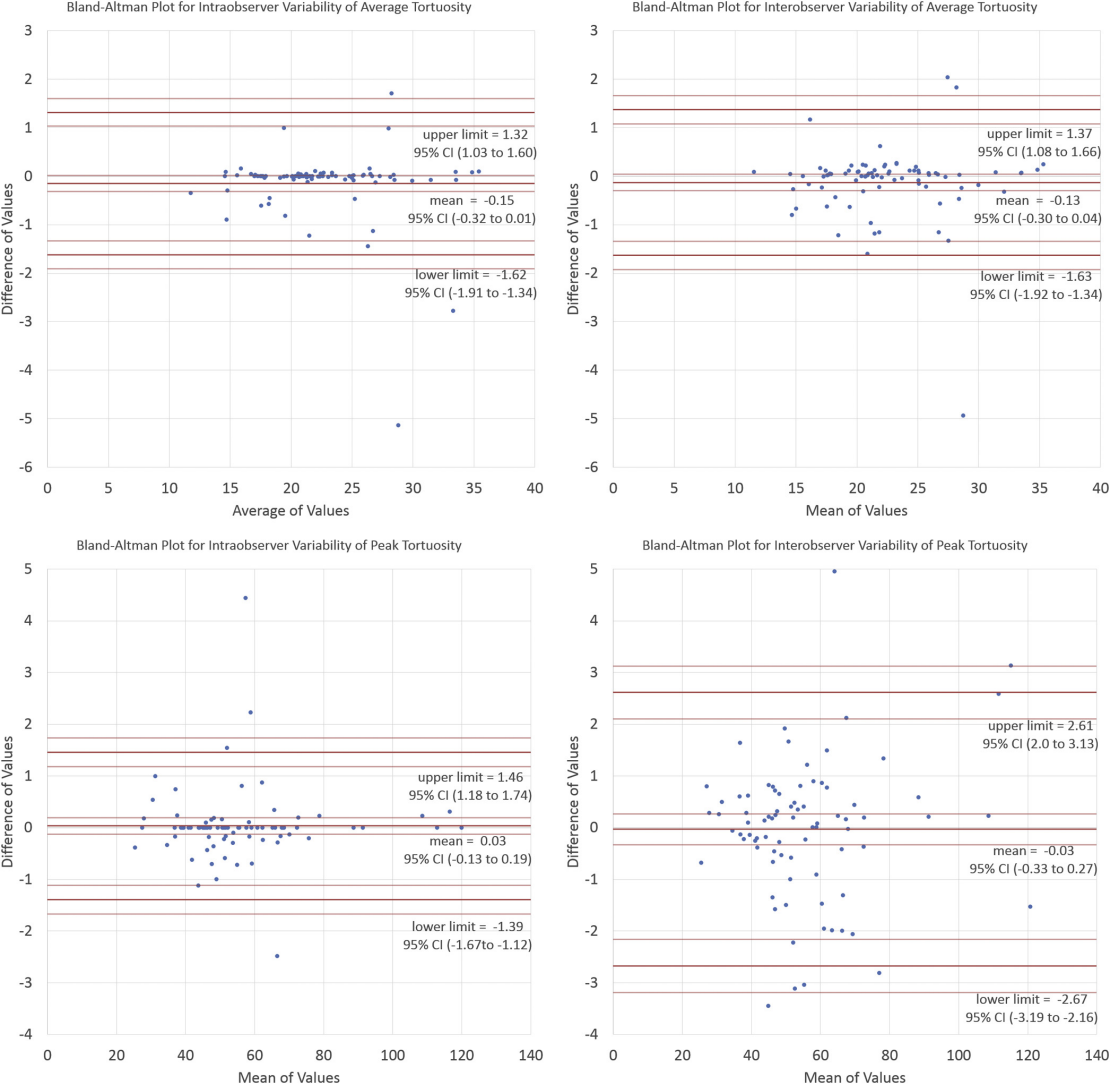
**The Bland-Altman plots are displayed with the mean values, the limits of agreement, and their 95% confidence intervals (CIs).** The vast majority of all points are within the limits of agreements for all graphs. There is low variability.

Low interobserver and intraobserver variability was observed for both the peak and average TI on repeat measurements using Bland-Altman plots (Figure 7). There was no appreciable difference between the classical ICC, the absolute agreement ICC, and the consistency ICC, suggesting that there was no bias between the different repeat measurements. All ICC scores were above 0.98, implying excellent reliability.

## Discussion

The Sentinel CEPD is used during TAVR to reduce the risk of stroke. Off-label uses have also been reported for other transcatheter procedures in which the risk of stroke is high.^11^ A contraindication for CEPD is excessive tortuosity, which was not clearly defined in the initial trial for the device. Although there are a variety of methods to define vessel tortuosity, a standardized definition has not been established. We present a novel TI based on preoperative CTA that may be a helpful tool to quantify vessel tortuosity and to determine whether a CEPD can be safely deployed.

Our proposed TI appropriately helps exclude patients with excessive tortuosity. Conversely, many patients with *visually* tortuous anatomy may actually have suitable anatomy for CEPD and should be offered the device. Our proposed TI cutoffs for safe CEPD delivery are a peak tortuosity of 74°/cm and an average tortuosity of 30°/cm.

Our study suggests that the peak tortuosity is a better assessment for CEPD suitability than average tortuosity. The patients in whom the CEPD could not be deployed successfully had a peak TI higher than the peak tortuosity cutoff. The average TI for two-thirds of the cases was below the proposed cutoff. This suggests that a high peak tortuosity would inhibit the use of Sentinel CEPD irrespective of the average tortuosity. This is because the peak tortuosity takes precedent over the average tortuosity as it is the most tortuous section of a vessel.

For the visually tortuous cohort of patients that did not have CEPD attempted, both the mean of the peak tortuosity and the mean of the average tortuosity were higher than for the patients in whom the CEPD was successfully deployed. However, the values for 7 of 14 (50.0%) patients in this cohort were still below the cutoff for both peak and average tortuosity. Therefore, these patients that were deemed to have excessively tortuous vessels might actually have been suitable for CEPD.

Though the Sentinel trial reported no adverse events from transradial implantation of CEPD, there is still a risk for transradial access site complications, stroke with manipulation in the great vessels, and a need for extra procedure time/contrast with CEPD placement. It is, therefore, important to incorporate a screening tool for CEPD eligibility to identify the patients in whom this device could be used. Future studies are required to determine the role of a TI on a larger number of patients and assess the utility of our TI in patients with tortuous anatomy.

### Limitations

Our study has limited data on tortuosity for patients in which CEPD was attempted and was unsuccessful (only 3 patients in total). Another limitation is that our proposed measure of tortuosity does not account for the effects of calcium deposits and aortic arch types. Furthermore, the first and last line segments are also excluded from our measure of tortuosity. Future studies are required to further evaluate if patients with visually excessive tortuosity are actually candidates for CEPD.

## Conclusion

Our proposed measure of tortuosity can be an important preoperative tool in determining a patient's suitability for placement of the Sentinel CEPD. This quantitative tool would help appropriately include and exclude patients for cerebral embolic protection.

## Ethics Statement

The research reported was conducted in accordance with the Declaration of Helsinki and has adhered to all relevant ethical guidelines. The study protocol was approved by the Northwell Health Institutional Review Board with a waiver of informed consent.

## Funding

The authors have no funding to report.

## Disclosure statement

Chad Kliger is a consultant and receives speaking honoraria from Siemens, Edwards Lifesciences, and Medtronic. The other authors had no conflicts to declare.
