# Supplementary material for: A Novel Computed Tomographic Angiography Tortuosity Index to Predict Successful Sentinel Cerebral Embolic Protection Delivery for Transcatheter Aortic Valve Replacement
Source: Struct Heart. 2022 Mar 31;6(2):100021. doi: 10.1016/j.shj.2022.100021 (PMC10236830; doi:10.1016/j.shj.2022.100021)
Supplement: Supplemental Table 1c [file mmc1.docx]

| x coordinate | y coordinate | z coordinate |
| --- | --- | --- |
| 22 | 321 | 72 |
| 77 | 167 | 86 |
| 198 | 216 | 157 |
| 264 | 273 | 202 |
| 364 | 309 | 208 |
| 436 | 290 | 184 |
| 472 | 243 | 121 |
| 495 | 200 | 60 |
| 521 | 165 | 16 |
| 552 | 143 | 3 |
| 603 | 127 | 23 |
| 632 | 151 | 114 |
| 649 | 178 | 168 |
| 686 | 212 | 214 |
| 739 | 237 | 174 |
| 785 | 239 | 137 |
| 842 | 224 | 128 |
| 904 | 217 | 186 |
| 930 | 225 | 235 |
| 933 | 252 | 299 |
| 912 | 311 | 385 |
| 921 | 341 | 463 |
| 943 | 351 | 546 |
| 967 | 351 | 631 |

**Supplemental Table 1C:** The x, y and z coordinates of the pixel location of the nodes are obtained from the snapshots in Supplemental Figures 1A and 1B. The x coordinate is repeated between the two snapshots and can be arbitrarily chosen to be all from the axial snapshot, Supplemental Figure 1A, or all from the coronal snapshot, Supplemental Figure 1B. This table uses the x coordinates of the axial snapshot in Supplemental Figure 1A. The data presented in this table and the scaling factor presented in Supplemental Figure 1A are used to calculate a peak tortuosity of 108.79 ⁰/cm and an average tortuosity 34.89 ⁰/cm for this subject.
