# Supplementary material for: A Novel Computed Tomographic Angiography Tortuosity Index to Predict Successful Sentinel Cerebral Embolic Protection Delivery for Transcatheter Aortic Valve Replacement
Source: Struct Heart. 2022 Mar 31;6(2):100021. doi: 10.1016/j.shj.2022.100021 (PMC10236830; doi:10.1016/j.shj.2022.100021)
Supplement: Supplemental Figure 1a [file mmc2.docx]

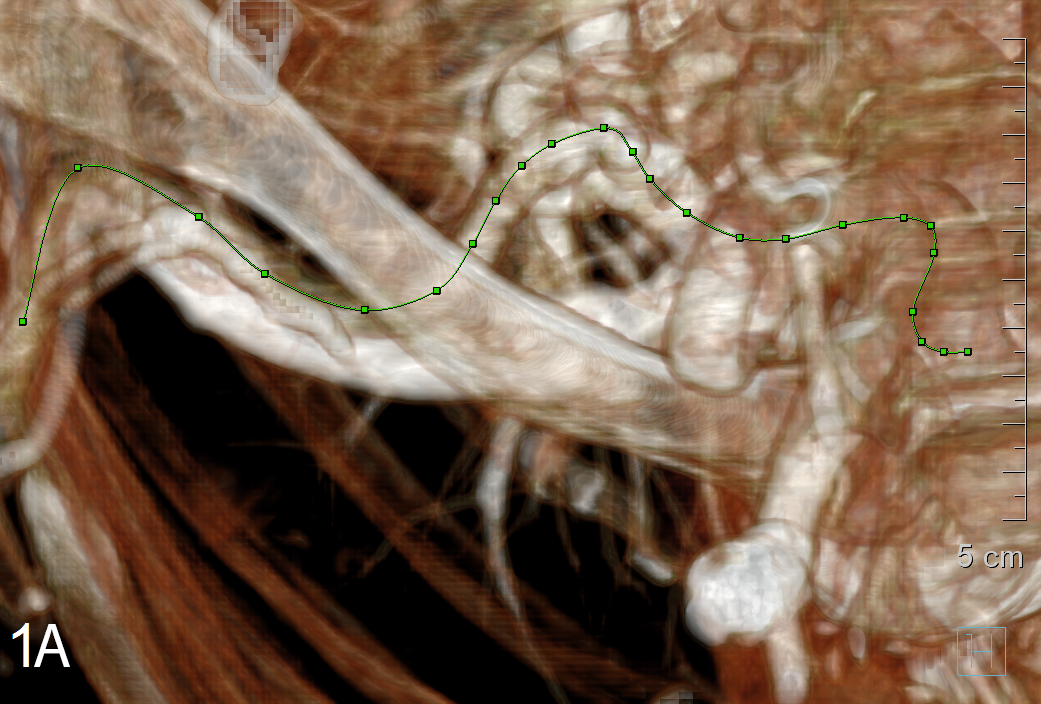


**Supplement Figure 1A:** The axial snapshot of the centerline from a subject from the unsuccessful cohort. When determining the pixel location of the nodes, the top left corner is chosen as the origin point with coordinate (0, 0). The horizontal axis is chosen for the x coordinate. The vertical axis is chosen for the y coordinate. The length of the scale presented in this figure is used to determine the scaling factor used to convert from pixels to centimeters. For this snapshot, the scaling factor is determined to be 96.6 pixel/cm.
