# Supplementary material for: A Novel Computed Tomographic Angiography Tortuosity Index to Predict Successful Sentinel Cerebral Embolic Protection Delivery for Transcatheter Aortic Valve Replacement
Source: Struct Heart. 2022 Mar 31;6(2):100021. doi: 10.1016/j.shj.2022.100021 (PMC10236830; doi:10.1016/j.shj.2022.100021)
Supplement: Supplemental Figure 1b [file mmc3.docx]

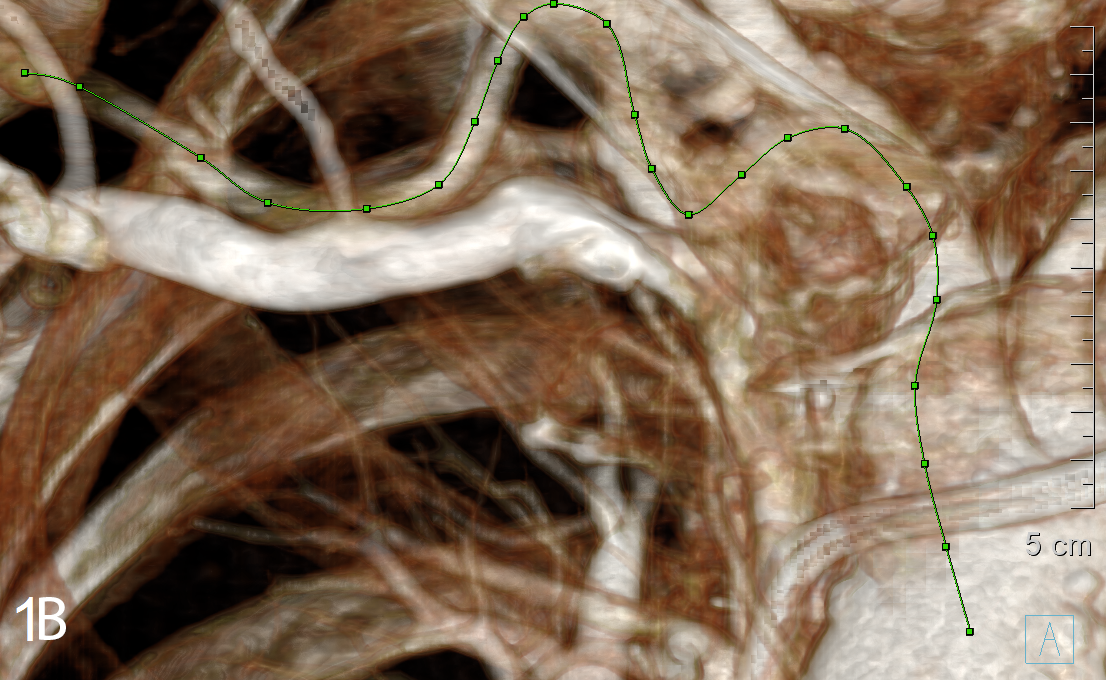


**Supplement Figure 1B:** The coronal snapshot of the centerline from the same subject as Supplemental Figure 1A. When determining the pixel location of the nodes, the top left corner is chosen as the origin point with coordinate (0, 0). The horizontal axis is chosen for the x coordinate. The vertical axis is chosen for the z coordinate. This snapshot is taken at the same magnification as Supplemental Figure 1A.
